# Association between composite dietary antioxidant index and fatty liver index among US adults

**DOI:** 10.3389/fnut.2024.1466807

**Published:** 2024-10-16

**Authors:** Meng Zheng, Chaochen Li, Jia Fu, Long Bai, Jinghui Dong

**Affiliations:** ^1^Department of Radiology, 5th Medical Center of Chinese PLA General Hospital, Beijing, China; ^2^Department of Radiology, Beijing Tongren Hospital Affiliated to Capital Medical University, Beijing, China

**Keywords:** CDAI, FLI, NAFLD, NHANES, cross-sectional survey

## Abstract

**Background:**

The potential beneficial health effects of dietary antioxidants have been reported. However, the association of a composite dietary antioxidant index (CDAI) with fatty liver index (FLI) remains unclear. This study aims to assess whether CDAI (including its components) is associated with FLI among US adults.

**Methods:**

This population-based cross-sectional study used data on US adults from the National Health and Nutrition Examination Survey (NHANES) 2007–2018 cycles. Weighted generalized linear regression models were used to analyze the association between CDAI (including vitamin A, C, E, zinc, selenium, and carotenoids) and FLI, which was calculated by using body mass index (BMI), waist circumference and levels of *γ*-glutamyl transferase and triglycerides.

**Results:**

Weighted generalized linear regression models showed an inverse association between CDAI and FLI in the total population (*β*, −0.40; 95% CI, −0.59, −0.21), in women (β, −0.56; 95% CI, −0.94, −0.18), and in men (β, −0.32; 95% CI, −0.54, −0.10) after adjusting for various confounders. The restricted cubic splines showed the negative linear dose–response associations between CDAI and FLI (all *P* non_linear >0.05). The dietary selenium intake in women has an inverse U-shaped relationship with FLI, with an inflection point value of 110 μg. In model 3, intake of dietary antioxidants Vitamins A, C, E, and carotenoids were significantly negatively associated with FLI in female but only were vitamins A and E negatively associated with FLI in male. In subgroup analysis, CDAI showed a significantly negative relation to FLI among those aged 60 years or older (*β*, −0.57; 95% CI, −0.81, −0.33), among those who engaged in active physical activity (*β*, −0.46; 95% CI, −0.63, −0.29), among those without metabolic syndrome (β, −0.43; 95% CI, −0.62, −0.24), and those without hyperuricemia (β, −0.43; 95% CI, −0.60, −0.26). Additionally, CDAI was significantly negatively associated with male FLI, regardless of whether they had diabetes or not.

**Conclusion:**

In conclusion, our results indicate that higher CDAI may be associated with a lower FLI.

## Introduction

The Fatty Liver Index (FLI) is a composite calculation integrating body mass index (BMI), waist circumference, triglyceride levels, and Gamma-Glutamyl Transferase (GGT) levels. It serves as a non-invasive biomarker for assessing hepatic steatosis in the absence of imaging or biopsy data. The FLI was initially designed to assist in identifying patients who may have Non-Alcoholic Fatty Liver Disease (NAFLD) (1–6), and has been validated as an effective tool for recognizing characteristics of liver fat accumulation associated with various metabolic disorders, such as insulin resistance, inflammation, and oxidative stress (7). Research has demonstrated that the FLI not only identifies individuals with liver fat accumulation but also predicts the risk of cardiovascular diseases (8–10). Notably, individuals with an FLI score of 60 or higher are considered to be at high risk for metabolic dysfunction-associated steatotic liver disease (MASLD) (8), which is closely related to the high risk of diabetes and cardiovascular diseases. Elevated FLI or hepatic steatosis is associated with an increased risk of cardiovascular diseases and dementia (11, 12). This indicates that the FLI is an important indicator for assessing the risk of multiple metabolic comorbidities. The correlation between the FLI and various metabolic syndrome parameters and adverse lipid profiles further confirms its suitability as an indicator for assessing the risk of metabolic syndrome (13). An increase in body weight and fasting blood glucose levels was associated with higher FLI values (14, 15). This further substantiates the FLI as an effective tool for assessing the risk of type 2 diabetes. FLI has also been found to be associated with a wide range of diseases in various systems, such as fractures (16, 17) hyperuricemia (18, 19), hypertension (20, 21), chronic kidney disease (22), lung cancer risk (23), and all-cause and cause-specific mortality (24). Therefore, it is crucial to find factors that can reduce FLI.

With the emphasis on healthy lifestyles, research on antioxidants in the diet is increasing, the composite dietary antioxidant index (CDAI), a composite score of multiple dietary antioxidants (including vitamin A, C, E, zinc, selenium, and carotenoids), represents an individual’s comprehensive dietary antioxidant intake profile, has been found to be associated with a variety of chronic diseases such as coronary heart disease, hypertension, chronic kidney disease, metabolic syndrome and hyperuricemia (25–29). Dietary antioxidants are known can firstly alleviate oxidative stress and reduce the occurrence of lipid peroxidation. Secondly, antioxidants can regulate lipid metabolic pathways, promote lipid oxidative metabolism and reduce fat accumulation in the liver. In addition, antioxidants have anti-inflammatory and antifibrotic effects. Some studies have reported the role of dietary antioxidants in fatty liver disease. A study found that MAFLD prevalence, CAP, HSI, and FLI, all decreased with increased daidzein intake, suggesting that daidzein intake may improve hepatic steatosis (30). Christensen K et al. found that higher intake and serum levels of most carotenoids were associated with lower odds of having NAFLD (31).

However, there are still many controversies and uncertainties about the mechanism and clinical application of dietary antioxidants on FLI. In this study, we investigated for the first time the independent and joint associations of CDAI (including vitamin A, vitamin C, vitamin E, zinc, selenium, and carotenoids) with FLI using a cross-sectional design. Based on previous studies, we hypothesize that there may be a potential negative correlation between CDAI and FLI, and that FLI decreases with increasing CDAI.

## Material and methods

### Data sources

NHANES is a nationally representative survey conducted by the National Center for Health Statistics (NCHS). It’s designed to assess the health and nutritional status of adults and children in the United States. The survey is unique in that it combines interviews and physical examinations (32). To select participants representative of the civilian, non-institutionalized US population, NHANES excluded all persons in supervised care or custody in institutional settings, all active-duty military personnel, active-duty family members living overseas, and any other US citizens residing outside the 50 states and the District of Columbia (33). Detailed survey operation manuals, consent documents, and brochures of each period are available on the NHANES website. NHANES was approved by the National Center for Health Statistics Institutional Review Board and all participants signed informed consent (32).

### Study design and population

**Figure 1:**
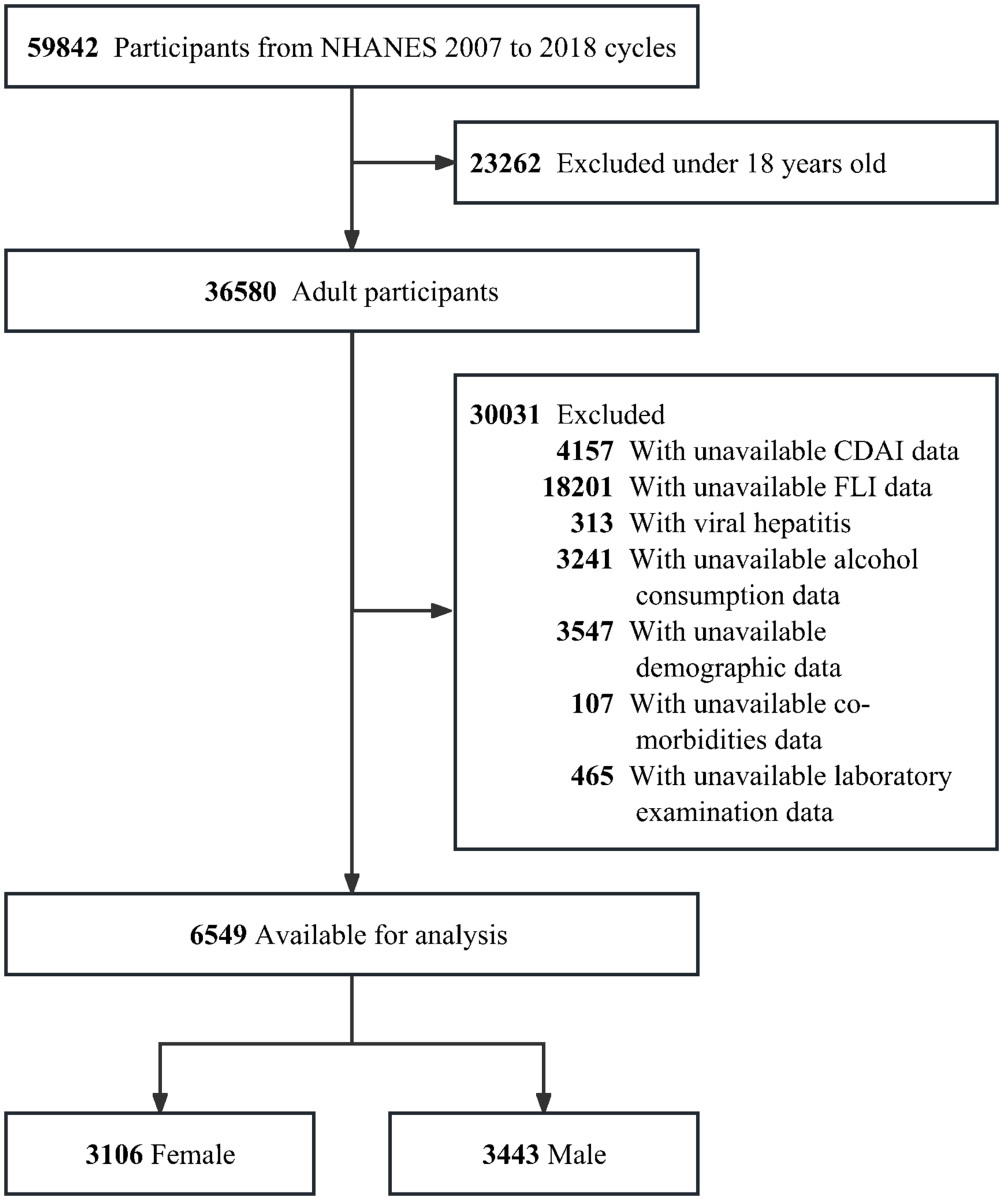
The flowchart of this study.

The NHANES 2007–2018 survey data were analyzed in this cross-sectional study. Participants under the age of 18 (*n* = 23,262), missing information on CDAI (*n* = 4,157), missing data to calculate the FLI (*n* = 18,201) and with viral hepatitis (*n* = 313) were all excluded. Furthermore, any missing data on alcohol consumption status (*n* = 3,241) were excluded, as were any missing data on demographic data of age, gender, race/ethnicity, education level, marital status, poverty-to-income (PIR), height, weight, blood pressure, total metabolic equivalent of physical activity (PA MET) and smoking status (*n* = 3,547), missing co-morbidities data on diabetes mellitus (DM), metabolic dysfunction (MetS), hypertension, cardiovascular disease (CVD), chronic kidney disease (CKD) and malignancy (*n* = 107), and missing blood biochemical indicators (alanine aminotransferase (ALT), aspartate aminotransferase (AST), uric acid (UA), platelets (PLT), albumin and total energy intake were further excluded (*n* = 465) (Figure 1).

### Definition

#### Fatty liver index

FLI was based on BMI, waist circumference, triglycerides and gamma-glutamyl-transferase (GGT) (34). The FLI formula is:

$$FLI=\left\{\left(\begin{array}{l} e^{0.953*\log_{e}\left(\text{triglycerides}\right)}+0.139*BMI+0.718*\\ \log_{e}\left(GGT\right)+0.053*\text{waist}\;c\text{ircumference}-15.745 \end{array}\right)/\left(\begin{array}{l} 1+e^{0.953*\log_{e}\left(\text{triglycerides}\right)}+0.139*BMI+0.718*\\ \log_{e}\left(GGT\right)+0.053*\text{waist}\;c\text{ircumference}-15.745 \end{array}\right)\right\}*100$$

#### Dietary assessment

To assess the joint exposure due to dietary antioxidant intake, we used the modified dietary antioxidant composite index (CDAI) developed by Wright et al. That is, normalization was performed for each of the six dietary antioxidants (vitamin A, vitamin C, vitamin E, zinc, selenium, and carotenoids) by subtracting the mean from the intake of each antioxidant and dividing by the standard deviation. Next, calculate the CDAI by adding the standardized dietary antioxidant intake (23, 35, 36).

$$\text{CDAI}=\sum_{i=1}^{n=6}\frac{\text{Individual}\;\text{Intake}-\text{Mean}}{SD}$$

### Covariates

Demographics, lifestyle factors and laboratory data were available through baseline household questionnaires and laboratory examination and used as covariates, including age (<40, 40–60, ≥60 years), sex (female and male), body mass index (BMI) calculated as weight divided by height squared (<25, 25–30 and ≥ 30 kg/m^2^), poverty income ratio (PIR) calculated by dividing household or individual income by poverty guidelines for the survey year (<1.3, 1.3–3.5, ≥3.5), race/ethnicity (Non-Hispanic White, Non-Hispanic Black, Mexican American and Others), marital status (married/living with partner, widowed/divorced/separated, and never married), education level (lower than high school, high school or equivalent and college or above), smoking status (smoked more than 100 cigarettes in life and smoke not at all now indicated former smoker, smoked moth than 100 cigarettes in life and smoke some days or every day indicated current smoker, and smoked less than 100 cigarettes in life indicated never smoker), alcohol drinking user was categorized as never (had <12 drinks in lifetime), mild (≤1 drink per day for women or ≤ 2 drinks per day for men on average over the past year), heavy drinking (≤2 drinks per day for women or ≤ 3 drinks per day for men on average over the past year), physical activity (active (total MET ≥600 min/week) and inactive (total MET <600 min/week)).

The detailed clinical and laboratory evaluation methods have been described previously (37–39).

Hypertension was defined as: (1) SBP ≥ 140mmhg and DBP ≥ 90mmhg in three blood pressure measurements; (2) have been told by a doctor or health professional that you have hypertension; (3) have ever used antihypertensive drugs. Diabetes was defined as: (1) Triglyceride (TG) ≥ 150 mg/dL, Serum total cholesterol (TC) ≥ 200 mg/dL, low-density lipoprotein (LDL) ≥130 mg/Dl, high-density lipoprotein (HDL) <40 mg/dL for male or < 50 mg/dL for female; (2) have ever used lipid-lowering drugs. CKD was defined as kidney structure or function abnormalities, present for >3 months, with implications for health (40). CVD included coronary heart disease, congestive heart failure, heart attack, stroke, and angina. Hyperuricemia was defined as serum uric acid levels in males >420umol/L (7 mg/dL) and in females >360umol/L (6 mg/dL), measured twice on different days with fasting after a normal purine diet. Metabolic syndrome, variously known also as syndrome X, insulin resistance, etc., was defined by WHO as a pathologic condition characterized by abdominal obesity, insulin resistance, hypertension, and hyperlipidemia.

### Statistical methods

Histogram distribution, Q-Q plot, and Kolmogorov–Smirnov test were employed to assess the normality of variables. Normally distributed continuous variables were reported as mean ± standard error (SE), while skewed continuous variables were presented as median with interquartile range (IQR). Categorical variables were expressed as frequencies and percentages. Paired t-test or Wilcoxon signed-rank test was utilized to compare paired factors within groups. Chi-square test or Fisher’s exact test was used for categorical variables, One-Way ANOVA test for normally distributed variables, and Kruskal-Wallis H test for skewed distributions to examine differences among different CDAI groups. Multiple comparisons were adjusted using the Bonferroni correction, Tukey’s method, or LSD method.

All analyzes in this study include sample weight calculation, clustering, and stratification. During the data analysis process, complex sampling weight calculation provided by the NHANES analysis guide is used, and comprehensive weight calculation and weighting processing are performed on valid sample data. Potential nonlinear associations between the CDAI and CDAI components (vitamin A, vitamin C, vitamin E, zinc, selenium, and carotenoids) and FLI were examined with restricted cubic splines, the median or curve inflection point was taken as the reference point. Univariate linear analysis was applied to identify FLI-related factors. Significant variables in the univariate linear regression analysis and variables thought to be confounders based on existing literature and clinical judgment were included in a weighted generalized linear regression model, which was used to analyze the linear relationship between CDAI (including continuous variables and quartile groups) and CDAI components (vitamin A, vitamin C, vitamin E, zinc, selenium, and carotenoids) and FLI. Weighted logistic regression model was used to analyze the relationship between CDAI and NAFLD and MASLD (NAFLD was defined as FLI ≥ 60 without heavy alcohol consumption, MASLD was defined as FLI ≥ 60). Three models were constructed for the analysis. Model 1 was adjusted for NHANES cycles, age, sex, race, education level, marital status, and poverty-income ratio (PIR). Model 2 included additional adjustments for physical activity, smoking status, alcohol consumption, DM, MetS, hypertension, CVD, CKD, and malignancy. Model 3 further included adjustments for PLT, ALT, AST, albumin, UA, and total energy intake.

For the continuous variable, we first converted it to a categorical variable according to the clinical cut point and then performed an interaction test. Tests for effect modification for those subgroup indicators were followed by the likelihood ratio test. Multicollinearity was tested using the variance inflation factor (VIF) method, with a VIF ≥ 5 indicating the presence of multicollinearity. All statistical analyses were carried out using R Statistical Software (Version 4.2.2, http://www.R-project.org, The R Foundation and nhanesR package) and Free Statistics Analysis Platform (Version 1.9.2 (41), Beijing, China, http://www.clinicalscientists.cn/freestatistics). Free Statistics is a software tool that offers user-friendly interfaces for common analyses and data visualization. It leverages R as the underlying statistical engine, with the graphical user interface (GUI) developed in Python. The platform facilitates reproducible analysis and interactive computing, enabling users to perform analyses with ease. Statistical significance was defined as a two-sided *p*-value <0.05.

## Results

### Baseline characteristics of participants grouped by CDAI quartiles

Among 6,549 adults included in this study, 3,106 were female with a median age of 45.25 years (standard error 0.43) and 3,443 were male with a median age of 43.70 years (standard error 0.37). The inclusion and exclusions criteria of the population are shown in Figure 1.

Table 1 shows the characteristics of the study participants grouped by CDAI quartiles.

**Table 1:** Characteristics of the study participants by the quartiles of composite dietary antioxidant index.

Variable	Total	Q1 (−7.262, −1.977)	Q2 (−1.977, 0.096)	Q3 (0.096, 2.682)	Q4 (2.682, 83.672)	*P* value
	*N* = 6,549	*N* = 1,638	*N* = 1,638	*N* = 1,636	*N* = 1,637	
CDAI	0.99 (0.08)	−3.36 (0.03)	−0.91 (0.02)	1.28 (0.02)	6.09 (0.15)	< 0.0001
Vitamin_A, μg	642.58 (11.42)	243.87 (5.12)	447.83 (6.74)	656.66 (7.67)	1140.15 (32.43)	< 0.0001
Vitamin_C, mg	80.00 (1.66)	29.98 (1.05)	56.52 (1.55)	80.50 (2.30)	142.79 (4.40)	< 0.0001
Vitamin_E, mg	9.08 (0.11)	4.20 (0.08)	7.01 (0.11)	9.23 (0.13)	14.91 (0.25)	< 0.0001
Zinc, mg	12.00 (0.12)	6.33 (0.10)	10.08 (0.13)	12.81 (0.18)	17.67 (0.25)	< 0.0001
Selenium, μg	120.82 (1.01)	69.37 (0.95)	102.47 (1.00)	128.89 (1.25)	172.51 (2.35)	< 0.0001
Carotenoids, μg	10212.97 (260.60)	2791.20 (85.59)	5793.02 (156.88)	9878.68 (250.00)	20808.12 (759.43)	< 0.0001
Age, years	44.44 (0.33)	43.26 (0.53)	44.89 (0.47)	45.24 (0.58)	44.21 (0.57)	0.03
Age Categories, *n* (%)						0.002
<40	2,666 (43.05)	690 (46.66)	653 (43.26)	636 (39.94)	687 (42.94)	
40–60	2,218 (36.56)	502 (33.99)	529 (33.36)	586 (38.38)	601 (39.86)	
> = 60	1,665 (20.40)	446 (19.35)	456 (23.38)	414 (21.68)	349 (17.20)	
Sex, *n* (%)						0.01
Female	3,106 (47.50)	839 (52.35)	750 (46.96)	760 (47.14)	757 (44.34)	
Male	3,443 (52.50)	799 (47.65)	888 (53.04)	876 (52.86)	880 (55.66)	
BMI, kg/m^2^	28.47 (0.13)	28.84 (0.29)	28.76 (0.22)	28.19 (0.19)	28.18 (0.22)	0.02
BMI categories, *n* (%)						0.07
<25	2061 (32.73)	500 (31.77)	493 (30.37)	515 (33.88)	553 (34.58)	
25–30	2,201 (33.54)	540 (32.93)	548 (32.58)	576 (35.15)	537 (33.36)	
> = 30	2,287 (33.73)	598 (35.30)	597 (37.06)	545 (30.97)	547 (32.06)	
SBP, mmHg	119.77 (0.29)	120.41 (0.51)	120.55 (0.49)	118.87 (0.55)	119.41 (0.49)	0.04
DBP, mmHg	70.15 (0.25)	69.73 (0.39)	69.72 (0.46)	69.95 (0.38)	71.10 (0.35)	0.02
Total energy intake, kcal	2255.76 (13.61)	1465.57 (16.39)	2010.50 (20.92)	2405.55 (20.85)	2989.64 (29.71)	< 0.0001
PLT, 1000 cells/uL	239.17 (1.04)	248.80 (2.24)	238.80 (1.87)	235.88 (1.72)	234.80 (1.74)	< 0.0001
Albumin, g/dL	4.28 (0.01)	4.25 (0.01)	4.29 (0.01)	4.28 (0.01)	4.29 (0.01)	0.10
AST, U/L	22.00 (19.00, 27.00)	22.00 (18.00, 27.00)	23.00 (19.00, 27.00)	22.00 (19.00, 26.00)	23.00 (19.00, 28.00)	< 0.0001
ALT, U/L	21.00 (16.00, 28.00)	20.00 (15.00, 28.00)	21.00 (17.00, 28.00)	21.00 (16.00, 27.00)	22.00 (17.00, 30.00)	< 0.0001
UA, mg/dL	5.49 (0.02)	5.55 (0.05)	5.57 (0.05)	5.38 (0.04)	5.46 (0.04)	0.01
Hyperuricemia, *n* (%)						< 0.001
No	5,383 (82.2)	1,291 (78.8)	1,326 (81)	1,387 (84.8)	1,379 (84.2)	
Yes	1,166 (17.8)	347 (21.2)	312 (19)	249 (15.2)	258 (15.8)	
PIR, *n* (%)						< 0.0001
<1.3	1766 (18.01)	568 (25.60)	441 (18.01)	382 (14.55)	375 (15.17)	
1.3–3.5	2,421 (34.43)	631 (37.02)	641 (38.12)	610 (33.89)	539 (29.39)	
> = 3.5	2,362 (47.55)	439 (37.38)	556 (43.87)	644 (51.56)	723 (55.44)	
Race/Ethnicity, *n* (%)						< 0.0001
White	3,006 (70.59)	676 (65.25)	771 (71.99)	792 (72.92)	767 (71.39)	
Black	1,231 (10.05)	373 (13.71)	302 (9.56)	266 (8.65)	290 (8.87)	
Mexican American	931 (7.55)	246 (8.26)	229 (7.25)	228 (7.11)	228 (7.69)	
Other	1,381 (11.80)	343 (12.78)	336 (11.20)	350 (11.32)	352 (12.04)	
Education level, *n* (%)						< 0.0001
Lower than high school	386 (2.80)	142 (4.47)	101 (2.83)	80 (2.49)	63 (1.70)	
High school or equivalent	2,152 (29.20)	649 (37.82)	575 (31.28)	470 (25.56)	458 (23.75)	
College or above	4,011 (68.00)	847 (57.72)	962 (65.89)	1,086 (71.95)	1,116 (74.56)	
Marital status, *n* (%)						0.001
Never married	1,396 (20.61)	378 (23.91)	342 (20.30)	330 (18.81)	346 (19.95)	
Married/Living with Partner	3,979 (64.64)	899 (58.20)	1,013 (64.96)	1,032 (67.46)	1,035 (66.85)	
Widowed/Divorced/Separated	1,174 (14.75)	361 (17.88)	283 (14.73)	274 (13.73)	256 (13.19)	
MET, min/week	4916.87 (135.22)	4862.71 (209.49)	4956.39 (251.27)	4851.08 (219.30)	5010.28 (230.42)	0.93
Physical Activity, *n* (%)						0.02
Active	5,411 (83.87)	1,310 (81.30)	1,360 (84.23)	1,342 (83.07)	1,399 (86.45)	
Inactive	1,138 (16.13)	328 (18.70)	278 (15.77)	294 (16.93)	238 (13.55)	
Smoke status, *n* (%)						< 0.0001
Never	3,784 (57.91)	876 (51.52)	938 (58.07)	977 (60.91)	993 (60.09)	
Former	1,510 (24.02)	348 (22.25)	390 (23.83)	389 (23.76)	383 (25.93)	
Now	1,255 (18.06)	414 (26.23)	310 (18.10)	270 (15.33)	261 (13.98)	
Alcohol user, *n* (%)						< 0.0001
Never	860 (10.02)	264 (12.23)	221 (11.02)	198 (9.21)	177 (8.05)	
Mild	2,801 (44.40)	597 (35.20)	717 (44.80)	732 (47.35)	755 (48.71)	
Heavy	2,888 (45.58)	777 (52.57)	700 (44.18)	706 (43.44)	705 (43.24)	
Hypertension, *n* (%)						0.14
No	4,195 (67.71)	984 (64.95)	1,058 (67.37)	1,088 (70.06)	1,065 (67.97)	
Yes	2,354 (32.29)	654 (35.05)	580 (32.63)	548 (29.94)	572 (32.03)	
DM, *n* (%)						0.06
No	5,539 (88.73)	1,353 (87.01)	1,368 (87.66)	1,406 (89.26)	1,412 (90.63)	
Yes	1,010 (11.27)	285 (12.99)	270 (12.34)	230 (10.74)	225 (9.37)	
MetS, *n* (%)						0.80
No	4,525 (71.12)	1,104 (70.59)	1,134 (70.30)	1,142 (72.18)	1,145 (71.29)	
Yes	2024 (28.88)	534 (29.41)	504 (29.70)	494 (27.82)	492 (28.71)	
CVD, *n* (%)						0.01
No	6,069 (94.25)	1,488 (93.39)	1,508 (93.31)	1,518 (94.06)	1,555 (96.02)	
Yes	480 (5.75)	150 (6.61)	130 (6.69)	118 (5.94)	82 (3.98)	
CKD, *n* (%)						0.03
No	5,723 (90.20)	1,383 (88.12)	1,433 (89.92)	1,436 (90.73)	1,471 (91.64)	
Yes	826 (9.80)	255 (11.88)	205 (10.08)	200 (9.27)	166 (8.36)	
Malignancy, *n* (%)						0.88
No	6,032 (91.55)	1,514 (92.21)	1,504 (91.52)	1,511 (91.26)	1,503 (91.33)	
Yes	517 (8.45)	124 (7.79)	134 (8.48)	125 (8.74)	134 (8.67)	
FLI	47.51 (0.63)	49.28 (1.03)	49.28 (1.22)	46.01 (1.02)	45.87 (1.21)	0.03

The characteristics of the participants were described using frequency (%), median (standard error) or median and interquartile range (IQR). CDAI = composite dietary antioxidant index, BMI = body mass index, PIR = poverty income ratio, SBP = systolic pressure, DBP = diastolic pressure, PLT = platelet count, ALT = alanine aminotransferase, AST = aspartate aminotransferase, UA = uric acid, MET = metabolic equivalent, DM = diabetes mellitus, MetS = metabolic syndrome, CVD = cardiovascular disease, CKD = chronic kidney disease, FLI = fatty liver index.

The four groups differed in sex, age, race, BMI, systolic pressure, diastolic pressure, poverty-to-income, education level, marital status, physical activity status, smoke status, alcohol user, cardiovascular disease, chronic kidney disease, total energy intake, platelet count, alanine aminotransferase, aspartate aminotransferase, uric acid and fatty liver index (all *p* value <0.05).

The highest CDAI quartile group tends to be married or living with partner, never-smoker, mild-drinker, active physical activity, with higher total energy intake, economic level and education level, with a lower BMI, platelet count and FLI. In contrast, the lowest CDAI quartile group tends to be were widowed, divorced or separated, current-smokers and never-drinkers, with CVD, CKD and low economic level, but there was no difference in hypertension, diabetes mellitus, metabolic syndrome and malignancy among different CDAI quartile groups.

**Figure 2:**
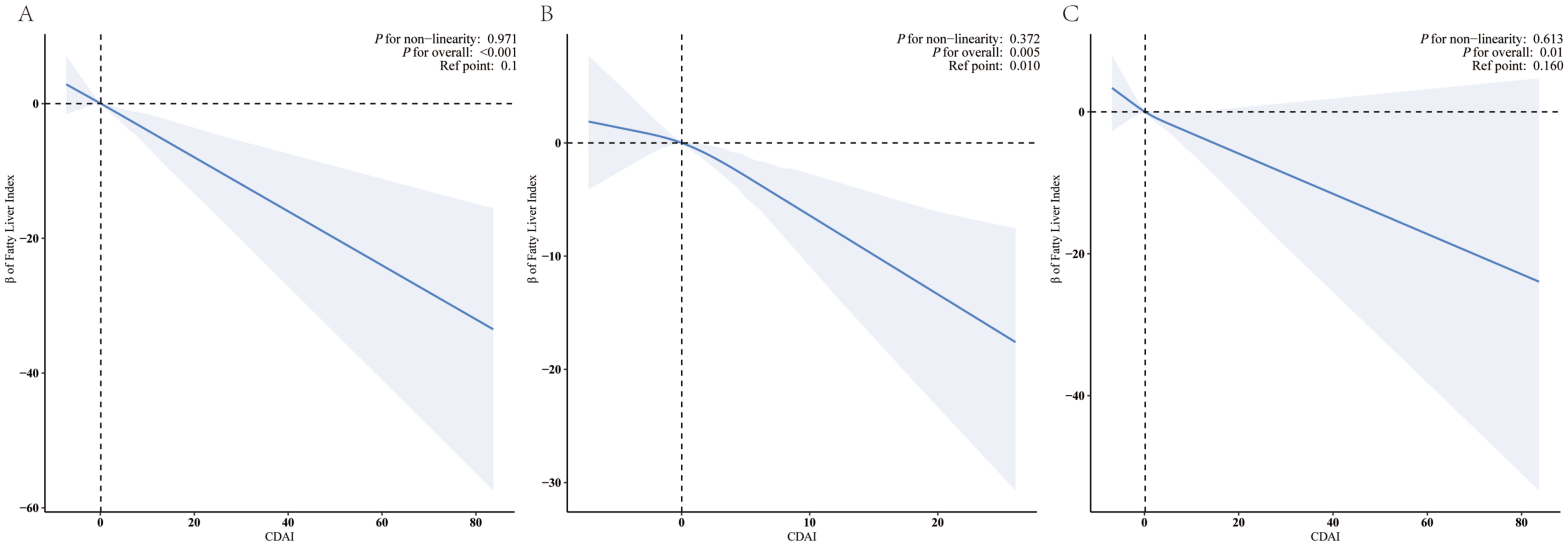
Restricted cubic splines for the relationships between CDAI and FLI. Figure 2 presents restricted cubic spline plots illustrating the relationship between Composite Dietary Antioxidant Index (CDAI) and the Fatty Liver Index (FLI) after adjusting for NHANES cycles, age, sex, race, education level, marital status, PIR, Physical Activity, smoke status, alcohol user, DM, MetS, Hypertension, CVD, CKD, malignancy, PLT, ALT, AST, albumin, UA, and total energy intake. The bold blue central lines represent the estimated adjusted effect value, with shaded ribbons indicating the 95% confidence intervals. The horizontal dashed lines represent the *β* value of 0.0 (reference point). The reference point was set at the median level of CDAI. The vertical dashed lines indicate the threshold value of CDAI at reference point. Please note the wide divergence in the 95% confidence intervals at the extremes due to the small number of patients and the cubic fit. (A) all participants, (B) female, (C) male.

Full model multivariable-adjusted restricted cubic spline analysis showed a “linear decrease” in the association of CDAI with FLI, and the negative linear dose–response association both between female CDAI and male CDAI and FLI (Figure 2, *P* for non-linearity = 0.971 for all participants, 0.372 for female and 0.613 for male).

### Factors associated with fatty liver index

Advanced age, male gender, Mexican-American, married or living with a partner, living alone, former-smoker, heavy-drinker, inactive physical activity, higher total energy intake, BMI, PLT, ALT, AST, UA values, and DM, hypertension, MetS-ATP, CKD, CVD disease were positively associated with FLI, whereas college or above education level, higher PIR, and albumin were negatively related to FLI (all *p* value <0.05) (Supplementary Table S1).

### Association between CDAI and FLI

In multivariable linear regression analyses, with adjustment for potential confounders (Table 2, model 3), Composite Dietary Antioxidant Index (DAI expressed as a continuous variable (per 1 unit) was decreased associated with FLI (*β* = −0.40, −0.59 ∼ −0.21, *p* < 0.001), as well as female CDAI (β = −0.56, −0.94 ∼ −0.18, *p* = 0.005) and male CDAI (β = −0.32-0.54 ∼ −0.10, *p* = 0.005) with FLI.

**Table 2:** Association between CDAI and FLI.

Subgroup	Crude model		Model 1		Model 2		Model 3	
	β (95% CI)	*P_*value	β (95% CI)	*P_*value	β (95% CI)	*P_*value	β (95% CI)	*P_*value
Overall (*N* = 6,549)								
CDAI (continuity value)	**−0.57 (−0.81, −0.32)**	**<0.001**	**−0.55 (−0.77, −0.34)**	**<0.001**	**−0.44 (−0.60, −0.29)**	**<0.001**	**−0.40 (−0.59, −0.21)**	**<0.001**
Q1 (−7.262, −1.977)	Reference		Reference		Reference		Reference	
Q2 (−1.977, 0.096)	0.01 (−3.06, 3.08)	0.995	−0.55 (−3.47, 2.36)	0.708	−0.15 (−2.77, 2.48)	0.911	0.43 (−1.88, 2.74)	0.712
Q3 (0.096, 2.682)	**−3.27 (−6.01, −0.52)**	**0.020**	**−3.51 (−6.15, −0.87)**	**0.010**	**−2.47 (−4.77, −0.16)**	**0.036**	−1.18 (−3.38, 1.01)	0.285
Q4 (2.682, 83.672)	**−3.40 (−6.55, −0.25)**	**0.035**	**−3.35 (−6.29, −0.41)**	**0.026**	**−3.23 (−5.51, −0.95)**	**0.006**	−2.51 (−5.08, 0.07)	0.056
*P* for trend		0.007		0.006		<0.001		0.022
Stratified by sex								
Female (*N* = 3,106)								
CDAI (continuity value)	**−0.91 (−1.26, −0.56)**	**<0.001**	**−0.66 (−1.00, −0.31)**	**<0.001**	**−0.40 (−0.67, −0.13)**	**0.004**	**−0.56 (−0.94, −0.18)**	**0.005**
Q1 (−7.262, −2.195)	−2.79 (−6.83, 1.26)	0.175	−2.65 (−6.16, 0.87)	0.138	−3.31 (−7.06, 0.44)	0.083	−2.43 (−5.60, 0.73)	0.129
Q2 (−2.195, 0.008)	Reference		Reference		Reference		Reference	
Q3 (0.008, 2.598)	**−8.46 (−13.03, −3.90)**	**<0.001**	**−3.83 (−7.36, −0.30)**	**0.034**	**−6.96 (−11.31, −2.60)**	**0.002**	**−3.11 (−6.03, −0.19)**	**0.038**
Q4 (2.598, 26.061)	**−8.03 (−11.72, −4.34)**	**<0.001**	**−3.98 (−7.21, −0.75)**	**0.017**	**−6.28 (−10.10, −2.46)**	**0.002**	**−4.92 (−8.16, −1.69)**	**0.003**
*P* for trend		<0.001		0.013		<0.001		0.003
Male (*N* = 3,443)								
CDAI (continuity value)	**−0.46 (−0.74, −0.18)**	**0.002**	**−0.49 (−0.76, −0.22)**	**<0.001**	**−0.48 (−0.71, −0.25)**	**<0.001**	**−0.32 (−0.54, −0.10)**	**0.005**
Q1 (−6.866, −1.821)	Reference		Reference		Reference		Reference	
Q2 (−1.821, 0.164)	−3.37 (−7.43, 0.68)	0.102	−2.79 (−6.28, 0.70)	0.115	−4.20 (−8.06, −0.34)	0.033	−1.26 (−4.03, 1.51)	0.366
Q3 (0.164, 2.753)	−1.32 (−5.78, 3.13)	0.557	−2.93 (−6.65, 0.78)	0.119	−2.75 (−7.14, 1.64)	0.216	−1.22 (−4.26, 1.83)	0.427
Q4 (2.753, 83.672)	−4.10 (−8.35, 0.16)	0.059	**−5.43 (−8.68, −2.17)**	**0.001**	**−4.69 (−8.82, −0.57)**	**0.026**	−3.16 (−6.43, 0.12)	0.059
*P* for trend		0.137		0.002		0.065		0.079

Crude model: adjusted for nothing. Model 1: adjusted for NHANES cycles, age, sex, race, education level, marital status, PIR. Model 2: adjusted for Model 1 and Physical Activity, smoke status, alcohol user, DM, MetS, Hypertension, CVD, CKD, and malignancy. Model 3: adjusted model 2 and PLT, ALT, AST, albumin, UA and total energy intake. The gender variable was no longer adjusted for stratification by sex. CDAI = composite dietary antioxidant, FLI = fatty liver index, CI = confidence interval, NHANES = National Health and Nutrition Examination Survey, PIR = poverty income ratio, CVD = cardiovascular disease, CKD = chronic kidney disease, PLT = platelet count, ALT = alanine aminotransferase, AST = aspartate aminotransferase, UA = uric acid, DM = diabetes mellitus, MetS = metabolic syndrome. AST = aspartate aminotransferase, UA = uric acid, DM = diabetes mellitus, MetS = metabolic syndrome. Results that are statistically significant are presented in bold font.

After adjusting for socioeconomic factors (model 1), the highest quarter (Q4) of CDAI (β = −3.35, −6.29 ∼ −0.41, *p* = 0.026) was significantly associated with decreased FLI compared with the first quarter (Q1), as well as in male (*β* = −5.43, −8.68 ∼ −2.17, *p* = 0.001), while the highest quarter of female CDAI (Q4) (*β* = −3.98, −7.21 ∼ −0.75, *p* = 0.017) was also significantly associated with decreased FLI compared with the second quarter (Q2).

The relationship remained valid after continued adjustment for factors such as lifestyle habits and comorbidities (model 2). However, after adjusting for blood indices and total energy intake, compared to the second quarter (Q2), the highest quarter (Q4) of female CDAI (*β* = −4.92, −8.16 ∼ −1.69, *p* = 0.003) was found to be significantly associated with decreased FLI, and the fourth quarter of total CDAI (β = −2.51, −5.08 ∼ 0.07, *p* = 0.056) and the male CDAI (β = −3.16, −6.43 ∼ 0.12, *p* = 0.059) were still associated with decreased FLI compared to the first quarter but were not statistically significant.

Weighted multivariable logistic regression analysis between CDAI and NAFLD and MASLD, showed that in the total population, CDAI was negatively correlated with both NAFLD and MASLD. For each unit increase in CDAI, the risk of NAFLD and MASLD was reduced by 5.7 and 3.4%, respectively (Model 3). After stratification by gender, this negative correlation still existed, but the results were not statistically significant, especially for female CDAI and NAFLD (Supplementary Table S2).

### Association between CDAI components and FLI

In the multivariable linear regression analyses, after adjusting for potential confounders as outlined in Table 3, model 3, the intake of vitamin A (*β* = −0.00174, −0.00297 ∼ −0.00051, *p* = 0.006), vitamin C (*β* = −0.01079, −0.01771 ∼ −0.00387, *p* = 0.003), vitamin E (β = −0.22052, −0.35073 ∼ −0.09031, *p* = 0.001), and carotenoids (β = −0.00007, −0.00011 ∼ −0.00003, *p* = 0.002), when expressed as a continuous variable (per 1 unit), was found to be inversely associated with FLI, as well as the female intake of vitamin A (β = −0.0029, −0.00505 ∼ −0.00075, *p* = 0.009), vitamin C (β = −0.02071, −0.03311 ∼ −0.00831, *p* = 0.001), vitamin E (β = −0.25533, −0.48910 ∼ −0.02157, *p* = 0.033), carotenoids (β = −0.00012, −0.00022 ∼ −0.00002, *p* = 0.023) and FLI, but only intake of vitamin A (β = −0.00145, −0.00260 ∼ −0.00030, *p* = 0.014) and vitamin E (β = −0.22428, −0.36397 ∼ −0.08458, *p* = 0.002) was found to be inversely associated with FLI in man.

**Table 3:** Association between CDAI components and FLI.

Value	Crude model	Model 1	Model 2	Model 3
	β (95% CI), *P*_value	β (95% CI), *P*_value	β (95% CI), *P*_value	β (95% CI), *P*_value
Overall (*N* = 6,549)				
Vitamin_A	**−0.00323 (−0.00491, −0.00156), <0.001**	**−0.00372 (−0.00527, −0.00218), <0.001**	**−0.00258 (−0.00389, −0.00128), <0.001**	**−0.00174 (−0.00297, −0.00051), 0.006**
Vitamin_C	**−0.02345 (−0.03358, −0.01332), <0.001**	**−0.02813 (−0.03820, −0.01806), <0.001**	**−0.01901 (−0.02769, −0.01033), <0.001**	**−0.01079 (−0.01771, −0.00387), 0.003**
Vitamin_E	**−0.28998 (−0.45918, −0.12079), <0.001**	**−0.38814 (−0.54658, −0.22971), <0.001**	**−0.2758 (−0.40136, −0.15024), <0.001**	**−0.22052 (−0.35073, −0.09031), 0.001**
Zinc	**0.21127 (0.06418, 0.35836), 0.005**	0.06228 (−0.05150, 0.17606), 0.279	−0.0042 (−0.09216, 0.08375), 0.924	0.02318 (−0.07425, 0.12061), 0.636
Selenium	**0.02446 (0.00767, 0.04125), 0.005**	0.00522 (−0.01037, 0.02081), 0.507	−0.00504 (−0.01757, 0.00748), 0.424	−0.00858 (−0.02310, 0.00594), 0.242
Carotenoids	**−0.00012 (−0.00018, −0.00005), <0.001**	**−0.00014 (−0.00020, −0.00007), <0.001**	**−0.00012 (−0.00016, −0.00007), <0.001**	**−0.00007 (−0.00011, −0.00003), 0.002**
Stratified by sex				
Female (*N* = 3,106)				
Vitamin_A	**−0.00597 (−0.00869, −0.00325), <0.001**	**−0.00481 (−0.00736, −0.00225), <0.001**	**−0.00285 (−0.00491, −0.00080), 0.007**	**−0.0029 (−0.00505, −0.00075), 0.009**
Vitamin_C	**−0.03491 (−0.05243, −0.01738), <0.001**	**−0.03439 (−0.05095, −0.01782), <0.001**	**−0.02759 (−0.04031, −0.01487), <0.001**	**−0.02071 (−0.03311, −0.00831), 0.001**
Vitamin_E	**−0.54582 (−0.79217, −0.29948), <0.001**	**−0.39891 (−0.63924, −0.15857), 0.001**	**−0.22115 (−0.43362, −0.00867), 0.042**	**−0.25533 (−0.48910, −0.02157), 0.033**
Zinc	−0.15799 (−0.47131, 0.15532), 0.319	0.03075 (−0.27320, 0.33469), 0.841	0.07272 (−0.13947, 0.28491), 0.496	0.00442 (−0.27888, 0.28771), 0.975
Selenium	−0.00168 (−0.03449, 0.03113), 0.919	0.01112 (−0.02139, 0.04364), 0.498	0.01259 (−0.01155, 0.03673), 0.302	−0.00046 (−0.02831, 0.02739), 0.974
Carotenoids	**−0.00034 (−0.00046, −0.00022), <0.001**	**−0.00027 (−0.00040, −0.00014), <0.001**	**−0.00018 (−0.00029, −0.00008), 0.001**	**−0.00012 (−0.00022, −0.00002), 0.023**
Male (*N* = 3,443)				
Vitamin_A	**−0.00312 (−0.00480, −0.00144), <0.001**	**−0.00343 (−0.00505, −0.00181), <0.001**	**−0.00254 (−0.00405, −0.00104), 0.001**	**−0.00145 (−0.00260, −0.00030), 0.014**
Vitamin_C	**−0.02471 (−0.03726, −0.01216), <0.001**	**−0.02573 (−0.03880, −0.01267), <0.001**	**−0.01662 (−0.02758, −0.00565), 0.004**	−0.00797 (−0.01700, 0.00107), 0.083
Vitamin_E	**−0.35424 (−0.54101, −0.16747), <0.001**	**−0.39564 (−0.57243, −0.21885), <0.001**	**−0.33728 (−0.47515, −0.19941), <0.001**	**−0.22428 (−0.36397, −0.08458), 0.002**
Zinc	0.06262 (−0.06638, 0.19162), 0.338	0.05613 (−0.06801, 0.18028), 0.371	−0.0382 (−0.13234, 0.05594), 0.421	0.02788 (−0.06015, 0.11591), 0.529
Selenium	−0.00636 (−0.02517, 0.01245), 0.504	0.00087 (−0.01670, 0.01845), 0.922	−0.01283 (−0.02711, 0.00145), 0.077	−0.01060 (−0.02640, 0.00519), 0.184
Carotenoids	−0.00006 (−0.00013, 0.00002), 0.161	−0.00008 (−0.00015, 0.00000), 0.060	**−0.00008 (−0.00013, −0.00002), 0.006**	−0.00004 (−0.00009, 0.00001), 0.094

Crude model: adjusted for nothing. Model 1: adjusted for NHANES cycles, age, sex, race, education level, marital status, PIR. Model 2: adjusted for Model 1 and Physical Activity, smoke status, alcohol user, DM, MetS, Hypertension, CVD, CKD, and malignancy. Model 3: adjusted model 2 and PLT, ALT, AST, albumin, UA and total energy intake. The gender variable was no longer adjusted for stratification by sex. CDAI = composite dietary antioxidant, FLI = fatty liver index, CI = confidence interval, NHANES = National Health and Nutrition Examination Survey, PIR = poverty income ratio, CVD = cardiovascular disease, CKD = chronic kidney disease, PLT = platelet count, ALT = alanine aminotransferase, AST = aspartate aminotransferase, UA = uric acid, MET = metabolic equivalent, DM = diabetes mellitus, MetS = metabolic syndrome. Results that are statistically significant are presented in bold font.

In order to investigate the presence of a dose–response relationship between CDAI components and the Fatty Liver Index (FLI), a smoothing function analysis was conducted (Supplementary Figures S1, S2). Following adjustments for potential confounding factors, a nonlinear association between female selenium and FLI was identified (*P* for nonlinearity = 0.006, as shown in Supplementary Figure S1). The zenith of FLI level was observed at 110 μg, as calculated using a two-piecewise linear regression model. To the left of the inflection point, there was an increase in the level of FLI as female selenium levels increased (β1 = 0.09550, 0.04560 ∼ 0.14539, *p* < 0.001). Conversely, on the right side of the inflection point, a different relationship was observed (β2 = −0.01699, −0.05665 ~ 0.02268, *p* = 0.401), but not statistically significant, suggesting an inverted U-shaped relationship between female selenium intake and FLI, at the same time, inflection point analysis shows that only when the dietary selenium intake of men is greater than or equal to 124.7 μg, did it have a statistically significant negative correlation with FLI, as shown in Table 4.

**Table 4:** Threshold effect analysis of selenium intake on fatty liver index (FLI).

Threshold of selenium	Adjusted β	95% CI	*P* value
Female intake of selenium			
<110 μg	0.09550	0.04560, 0.14539	**<0.001**
≥110 μg	−0.01699	−0.05665, 0.02268	0.401
Likelihood ratio test			**<0.001**
Male intake of selenium			
<124.7 μg	−0.02123	−0.06853, 0.02606	0.379
≥124.7 μg	−0.02378	−0.04396, −0.00361	**0.021**
Likelihood ratio test			0.539

Adjusted for NHANES cycles, age, race, education level, marital status, PIR, Physical Activity, smoke status, alcohol user, DM, MetS, Hypertension, CVD, CKD, and malignancy, PLT, ALT, AST, albumin, UA and total energy intake. CI = confidence interval. Results that are statistically significant are presented in bold font.

### Subgroup analysis

Possible modifications of the association between the CDAI and FLI were evaluated for the following subgroups: age (<40, 45–60 vs. ≥60 years), physical activity (active vs. inactive), DM (no vs. yes), MetS-ATP (no vs. yes), and hyperuricemia (no vs. yes). There were statistically significant interaction effects between the physical activity, MetS and hyperurucemia groups (*p*-values for interactions were 0.008, <0.001 and 0.024, respectively) for total sample. Furthermore, after stratifying by gender, our analysis revealed similar results across the female metabolic syndrome and hyperuricemia groups in relation to FLI, with a statistically significant interaction observed (*p* = 0.003 and 0.041, respectively), as well as male physical activity and metabolic syndrome groups (*p* = 0.003 and 0.004, respectively). Men who did not engage in active physical activity showed an increase in FLI levels even with increased CDAI intake. However, the impact of physical activity on the relationship between CDAI and FLI still needs further validation. Conversely, no significant interactions were detected among the age and DM groups in relation to FLI (all *p* values for interaction >0.05), as shown in Supplementary Table S3.

## Discussion

In this large population-based cross-sectional study, we found a negative correlation between CDAI and FLI, and gender subgroup analysis showed that this phenomenon was more significant in the female population. Additionally, the study confirmed a negative correlation between dietary vitamin A, vitamin C, vitamin E, carotenoids, and FLI. Subgroup analysis by gender also revealed a negative correlation between these dietary antioxidants and FLI in females, while in males, a significant negative correlation was found only between vitamin A, vitamin E, and FLI. Importantly, we have identified an inverted U-shaped relationship between female dietary selenium intake and FLI (*P* nonlinearity = 0.006), with the peak level observed at 110 μg. Furthermore, our study found physical activity in men mediates the association between CDAI and FLI. Subsequent subgroup analysis within the age and diabetes mellitus groups showed that there were no significant interactions between the subgroups.

To date, few studies have reported the association between CDAI and FLI. This study is the first cross-sectional survey using a composite dietary antioxidant index to explain changes in the level of FLI. Compared to traditional dietary antioxidant measures, CDAI is significantly associated with many adverse health outcomes and has been demonstrated to have advantages and effectiveness in epidemiological studies. A cross-sectional study (42) highlighted a negative non-linear association between CDAI and depression in a nationally representative sample of US adults, before the inflection point of 0.16, each unit increase in CDAI was associated with a 30% decrease in the risk of depression, after the inflection point, the risk of depression was found to be reduced by 11% for each unit increase. Another cross-sectional assessment in the Kardiovize study (35) found CDAI negatively associates with carotid intima media thickness in women but not in men. Evidence from a Cancer Screening Trial (43) indicated a trend for a higher quartile of food-based CDAI (fCDAI) associated with a lower lung cancer risk after adjusting for covariates (HR (Q4vs.Q1) = 0.64, 95% CI: 0.52, 0.79; *P* for trend <0.001). A study (44) of middle-aged and elderly Americans found that CDAI was linearly negatively associated with depression [0.77 (0.67, 0.89)] and non-linearly negatively associated with all-cause mortality [0.91 (0.83, 1.00)] with an inflection point of-0.19. Another prospective cohort study (45) revealed a linear relationship between CDAI and all-cause mortality (0.97 (0.87–1.07) for Q2, 0.88 (0.81–0.96) for Q3, and 0.90 (0.80–1.00) for Q4 (*P* for trend = 0.009) upon comparison with the lowest quartile of CDAI), and an identical trend was observed for cardiovascular mortality. Therefore, this study utilized this indicator.

Our study utilized CDAI as a primary measure, uncovering a negative association with the FLI. Considering FLI’s role in diagnosing NAFLD and its link to metabolic diseases, we further examined the correlation between CDAI and NAFLD (defined as FLI ≥ 60 without excessive alcohol intake) as well as MASLD (defined as FLI ≥ 60). Our findings indicated an inverse relationship between CDAI and the prevalence of both NAFLD (OR: 0.943, 95% CI: 0.902, 0.987) and MASLD (OR: 0. 966, 95% CI: 0. 936, 0. 998). A cross-sectional study (46) involving 12,286 participants, after adjusting for age, gender and ethnicity, revealed a negative association between MASLD status and CDAI (OR: 0. 976, 95% CI: 0. 960, 0. 993), which is consistent with our findings. Another study (47) based on NHANES data from 2017 to 2020 found that compared to the first quartile of CDAI, the odds ratios for MASLD were 0.96 (95% CI: 0.82, 1.12) in the second quartile, 0.80 (95% CI: 0.68, 0.95) in the third quartile, and 0.60 (95% CI: 0.49, 0.73) in the fourth quartile, respectively, once again corroborating the results of our study. The above results suggest that CDAI played a significant role in improving FLI and its associated metabolic diseases.

In addition, our study also conducted an association analysis between the representative components of CDAI and FLI. This study confirmed that dietary antioxidants vitamin A, vitamin C, vitamin E, and carotenoids all have a positive contribution to the improvement of FLI. In a Korean NHANES study (48), vitamin A, vitamin C, and vitamin E were not significantly different between MASLD and non-MASLD patients. However, the majority of the literature still supports our conclusion.

The following related studies provide further support for the findings of this study.

Guo et al. (49) found serum vitamin C was negatively correlated with the risk of non-alcoholic fatty liver disease (defined as the US Fatty Liver Index ≥30 in the absence of other chronic liver disease) when its level was less than 0.92 mg/dL. Mazidi et al. (50) showed that when levels of vitamin A changed from low (1.53) to high (1.95), the fatty liver index (FLI) in the low BMI category changed from 36.1 to 24.3. Ivancovsky-Wajcman et al. (51) found that both vitamin A and E were related with the level of steatosis according to SteatoTest, their intake may be protective from NAFLD-related liver damage. All of these studies suggest that antioxidants have a protective effect on FLI, which is consistent with our findings that the intake of dietary vitamins A, C, E, carotenoids and CDAI can reduce FLI. But the protective role of dietary antioxidants in fatty liver disease still needs further verification.

Our research results also showed that regardless of gender, compared to other antioxidants, vitamin E has the most significant effect in reducing FLI. The potential mechanisms between vitamin E and fatty liver index mainly involve its antioxidant and anti-inflammatory effects. Oxidative stress plays a central role in the pathogenesis of fatty liver disease (FLD). As a potent antioxidant, vitamin E can reduce oxidative stress (52), thereby slowing down the progression of FLD.

In clinical trials, vitamin E has shown a positive effect on improving biochemical indicators (such as alanine aminotransferase (ALT) and aspartate aminotransferase (AST) levels) and hepatic pathological features in patients with NAFLD (53–57). Vitamin E can significantly improve steatosis, lobular inflammation, and ballooning scores in patients with non-alcoholic steatohepatitis (NASH) (58). Vitamin E also improves lipid metabolism in mice with non-alcoholic fatty liver disease through the Nrf2/CES1 signaling pathway (59) and improves liver function, lipid metabolism, and oxidative stress in rats with NAFLD induced by a high-fat, high-cholesterol diet (HFD) (59, 60). However, some studies suggest that vitamin E therapy can improve blood lipids to some extent, but its effect on children’s liver function and liver tissue is not apparent (61). Another study found vitamin E is not recommended for the treatment of diabetes, NAFLD without liver biopsy, NASH cirrhosis, or cryptogenic cirrhosis with NASH (62). Dietary intake of vitamin E proves advantageous in the prevention of NAFLD based on CAP threshold values of 288 dB/m and 263 dB/m, particularly among individuals devoid of hyperlipidemia (63). Although our research also found the beneficial effects of vitamin E in reducing the FLI, its efficacy still needs to be further verified in different populations.

Interestingly, we discovered an inverse U-shaped relationship between female dietary selenium intake and FLI. When the dietary selenium intake of women is less than 110 μg, the two are positively correlated, but as the selenium intake increases, the FLI decreases, but not statistically significant. Selenium is an essential trace element that plays a crucial role in human health, many studies have found a close relationship between selenium intake in the diet and liver fat. However, there has been ongoing controversy.

Consistent with our study, a cross-sectional study (64) based on baseline data from the prospective PERSIAN Kavar cohort study found that after adjusting for sociodemographic variables, smoking status, alcohol consumption, physical activity, and dietary factors, the odds ratios (ORs) for FLI-defined NAFLD in the fourth and fifth quintiles were 1.31 (95% confidence interval (CI): 1.01–1.70) and 1.50 (95% CI: 1.13–1.99), respectively, compared to the first quintile (*P* trend = 0.002). This result revealed a weak positive correlation between dietary selenium intake and the risk of NAFLD. Another study based on the NHANES database from 2017 to 2018 found that higher blood selenium levels (>205.32, ≤453.62 μg/L) were significantly positively correlated with NAFLD (ORs = 1.31). Additionally, men with high blood selenium levels showed a significant negative correlation with late-stage liver fibrosis (ORs = 0.61). But NAFLD and liver fibrosis are caused by an imbalance of selenium homeostasis, not by dietary selenium intake (65). These contradictory results have also been observed in previous animal studies. Steinbrenner et al. (66) found that severe selenium overdose and severe selenium deficiency could both alter animal liver metabolism. Dietary selenium levels above normal levels did not enhance the biosynthesis of liver selenoproteins or the activity of key selenium enzymes, but at high doses, reactive selenium metabolites might be produced, which could interfere with signal transduction or metabolic pathways. As a result, multiple synthetic metabolic pathways and lipid accumulation might be enhanced. Conversely, thebiosynthesis/activity of the major antioxidant selenoproteins was inhibited by dietary selenium deficiency, leading to oxidative stress and inflammation, which was associated with increased glucose and glutamine catabolic metabolism and reduced lipid accumulation. Taking into account that the dietary selenium intake is between 55-75 μg/d, with a maximum safe intake of 400 μg/d (67), and the median selenium intake of the population included in this study is 110 μg/d, within this intake level, selenium does promote FLI, in summary, sufficient dietary selenium intake can affect the deposition and metabolism of liver fat through various pathways, thereby playing a positive role in liver health. However, it is worth noting that excessive selenium intake may also have adverse effects on the liver. Therefore, selenium intake in the diet should be moderate to maintain liver health.

Our study also revealed that physical activity in men reverse the relationship between CDAI and FLI. While an increase in CDAI leads to a reduction in FLI in men, this effect is limited to those engaging in active physical activity. For men with inactive physical activity, CDAI is positively correlated with FLI. This finding suggests that even with active supplementation of dietary antioxidants, it may not alter fat deposition in men with low physical activity. Of course, further validation of this study is still needed. At the same time, our research found that participants with metabolic syndrome and hyperuricemia, increasing dietary CDAI intake did not reduce FLI, which further suggests the harmful effects of metabolic syndrome and hyperuricemia on obese individuals.

Given that FLI is associated with various diseases and adverse health outcomes, it is an important public health issue to improve it. Today, with the awakening of public health awareness, our research is conducted in this context, which is of great significance for guiding obese patients to have a healthy diet. Our research has found that dietary antioxidants play an important role in improving FLI, and has detailed how to choose suitable dietary antioxidants for different genders. Though, some of the findings in this study currently lack theoretical support, and we will further explore them.

However, there are still some limitations in this study. Firstly, cross-sectional design cannot establish the causal relationship between dietary antioxidant intake and the risk of FLI. Secondly, because of the nature of the NHANES database, the dietary questionnaire information was self-reported, which might introduce recall bias. Thirdly, this study tried to control confounding factors that may affect CDAI and FLI, but based on source data and existing theoretical limitations, it is not possible to include all control variables that might have influenced the results of the study. Finally, in this study, the reasons and mechanisms behind the gender differences in dietary antioxidants and FLI have not yet found effective theoretical support. It is necessary to further investigate the relevant physiological mechanisms in the future.

## Conclusion

This cross-sectional study reveals a significant inverse CDAI (including vitamin A, C, E) and the FLI, with dietary antioxidants showing potential in mitigating FLI, particularly in females. Female selenium intake and FLI showed a U-shaped relationship, suggesting an optimal level of selenium intake could help prevent fatty liver. The findings underscore the importance of dietary interventions in public health strategies to combat metabolic dysfunction-associated steatotic liver disease (MASLD). While the benefits of higher CDAI on reducing FLI may be modified by physical activity, metabolic syndrome, and hyperuricemia, highlighting the complexity of the relationship between diet, lifestyle, and liver health. Future research employing prospective study designs is warranted to establish causality and further elucidate the mechanisms underlying these associations.

## Data Availability

The datasets presented in this study can be found in online repositories. The names of the repository/repositories and accession number(s) can be found in the article/Supplementary material.

## Supplementary material

SUPPLEMENTARY FIGURE S1 Restricted cubic splines for the relationships between female dietary vitamin A, vitamin C, vitamin E, zinc, selenium, and total carotenoids intake and FLI. Figure S1 presents restricted cubic spline plots illustrating the relationship between female dietary vitamin A (A), vitamin C (B), vitamin E (C), zinc (D), selenium (E) and total carotenoids intake (F) and the Fatty Liver Index (FLI) after adjusting for NHANES cycles, age, race, education level, marital status, PIR, Physical Activity, smoke status, alcohol user, DM, MetS, Hypertension, CVD, CKD, malignancy, PLT, ALT, AST, albumin, UA, and total energy intake. The bold blue central lines represent the estimated adjusted effect value, with shaded ribbons indicating the 95% confidence intervals. The horizontal dashed lines represent the β value of 0.0 (reference point). The reference point was set at the median level of female CDAI components. The vertical dashed lines indicate the threshold value of female CDAI components at reference point. Please note the wide divergence in the 95% confidence intervals at the extremes due to the small number of patients and the cubic fit.

SUPPLEMENTARY FIGURE S2 Restricted cubic splines for the relationships between male dietary vitamin A, vitamin C, vitamin E, zinc, selenium, and total carotenoids intake and FLI. Supplementary Figure S2 presents restricted cubic spline plots illustrating the relationship between male dietary vitamin A (A), vitamin C (B), vitamin E (C), zinc (D), selenium (E) and total carotenoids intake (F) and the Fatty Liver Index (FLI) after adjusting for NHANES cycles, age, race, education level, marital status, PIR, Physical Activity, smoke status, alcohol user, DM, MetS, Hypertension, CVD, CKD, malignancy, PLT, ALT, AST, albumin, UA, and total energy intake. The bold blue central lines represent the estimated adjusted effect value, with shaded ribbons indicating the 95% confidence intervals. The horizontal dashed lines represent theβvalue of 0.0 (reference point). The reference point was set at the median level of male CDAI components. The vertical dashed lines indicate the threshold value of male CDAI components at reference point. Please note the wide divergence in the 95% confidence intervals at the extremes due to the small number of patients and the cubic fit.
