# Supplementary figures and images for: Association between composite dietary antioxidant index and fatty liver index among US adults
Source: Front Nutr. 2024 Oct 16;11:1466807. doi: 10.3389/fnut.2024.1466807 (PMC11521932; doi:10.3389/fnut.2024.1466807)

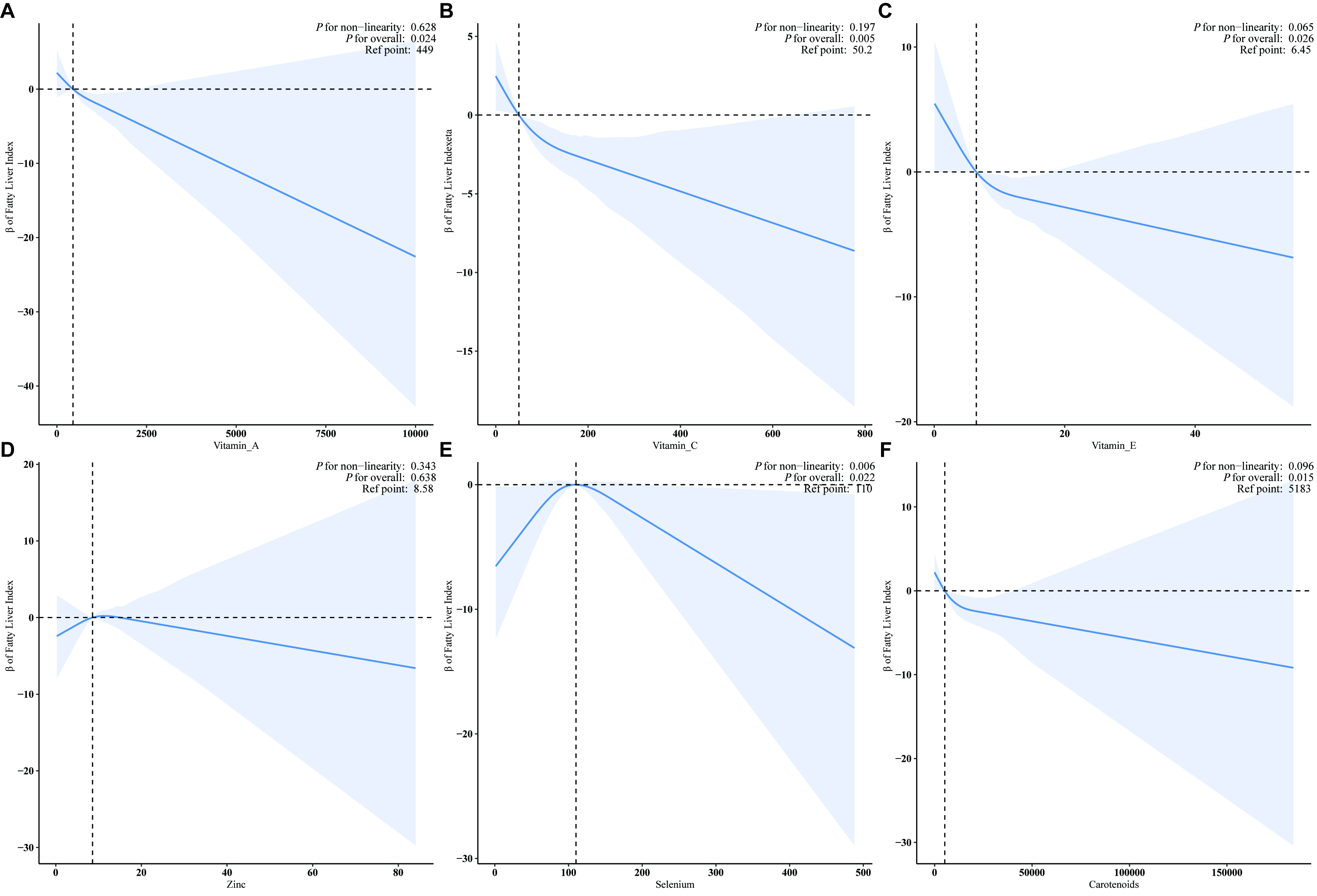

Supplement: SUPPLEMENTARY FIGURE S1 — Restricted cubic splines for the relationships between female dietary vitamin A, vitamin C, vitamin E, zinc, selenium, and total carotenoids intake and FLI. Figure S1 presents restricted cubic spline plots illustrating the relationship between female dietary vitamin A (A), vitamin C (B), vitamin E (C), zinc (D), selenium (E) and total carotenoids intake (F) and the Fatty Liver Index (FLI) after adjusting for NHANES cycles, age, race, education level, marital status, PIR, Physical Activity, smoke status, alcohol user, DM, MetS, Hypertension, CVD, CKD, malignancy, PLT, ALT, AST, albumin, UA, and total energy intake. The bold blue central lines represent the estimated adjusted effect value, with shaded ribbons indicating the 95% confidence intervals. The horizontal dashed lines represent the β value of 0.0 (reference point). The reference point was set at the median level of female CDAI components. The vertical dashed lines indicate the threshold value of female CDAI components at reference point. Please note the wide divergence in the 95% confidence intervals at the extremes due to the small number of patients and the cubic fit. [file Image_1.tif]

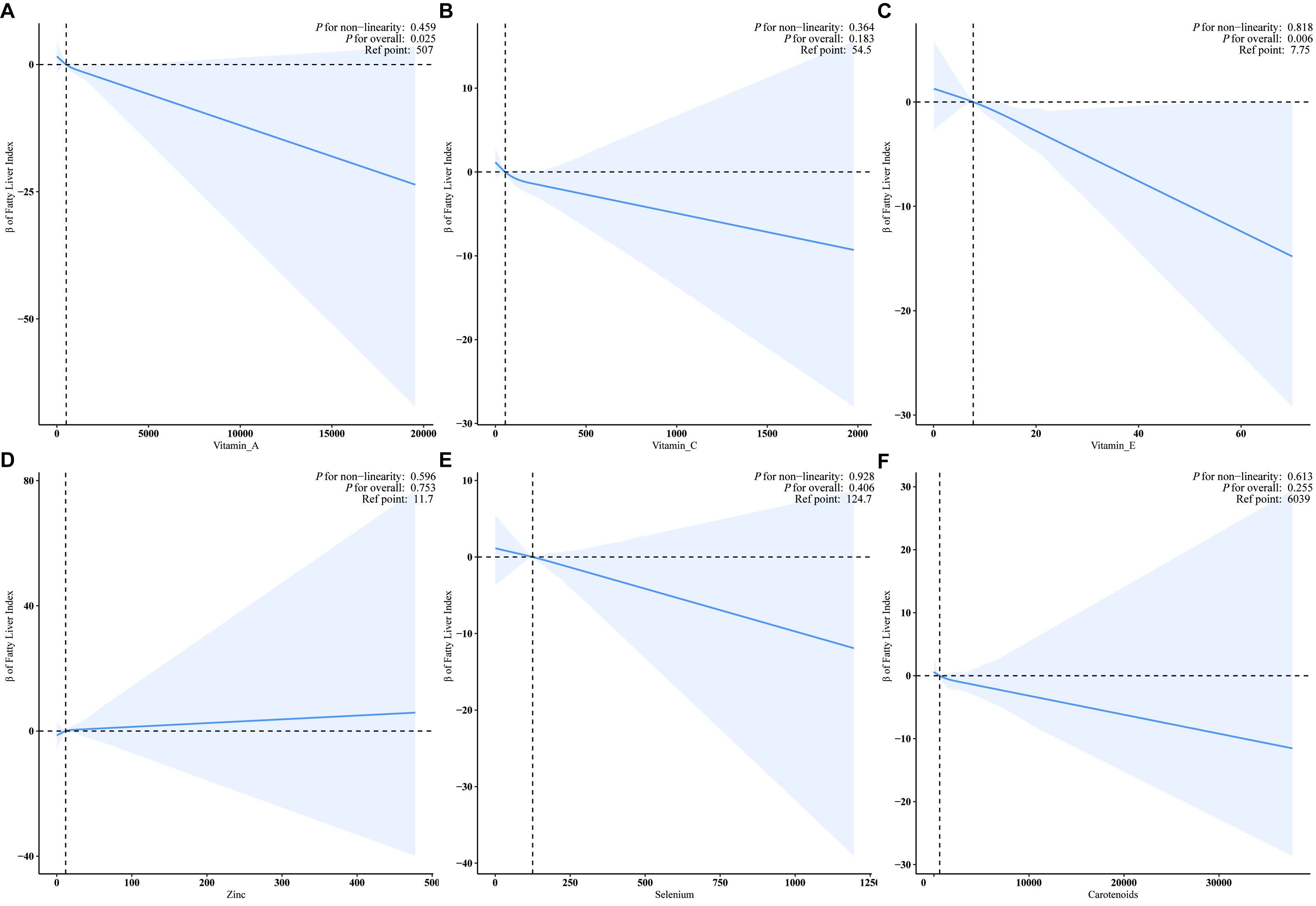

Supplement: SUPPLEMENTARY FIGURE S2 — Restricted cubic splines for the relationships between male dietary vitamin A, vitamin C, vitamin E, zinc, selenium, and total carotenoids intake and FLI. Supplementary Figure S2 presents restricted cubic spline plots illustrating the relationship between male dietary vitamin A (A), vitamin C (B), vitamin E (C), zinc (D), selenium (E) and total carotenoids intake (F) and the Fatty Liver Index (FLI) after adjusting for NHANES cycles, age, race, education level, marital status, PIR, Physical Activity, smoke status, alcohol user, DM, MetS, Hypertension, CVD, CKD, malignancy, PLT, ALT, AST, albumin, UA, and total energy intake. The bold blue central lines represent the estimated adjusted effect value, with shaded ribbons indicating the 95% confidence intervals. The horizontal dashed lines represent theβvalue of 0.0 (reference point). The reference point was set at the median level of male CDAI components. The vertical dashed lines indicate the threshold value of male CDAI components at reference point. Please note the wide divergence in the 95% confidence intervals at the extremes due to the small number of patients and the cubic fit. [file Image_2.tif]
